# BDNF belongs to the nurse-like cell secretome and supports survival of B chronic lymphocytic leukemia cells

**DOI:** 10.1038/s41598-020-69307-1

**Published:** 2020-07-28

**Authors:** Hugo Talbot, Sofiane Saada, Elodie Barthout, Paul-François Gallet, Nathalie Gachard, Julie Abraham, Arnaud Jaccard, Danielle Troutaud, Fabrice Lalloué, Thomas Naves, Anne-Laure Fauchais, Marie-Odile Jauberteau

**Affiliations:** 1 Equipe Accueil 3842 CAPTuR, Faculty of Medicine, Limoges University, 2, Rue du Docteur Marcland, 87025 Limoges Cedex, France; 2 Hematology Laboratory, Dupuytren Hospital University Center of Limoges, Limoges Cedex, France; 3 CNRS-UMR 7276, Limoges University, Limoges Cedex, France; 4 Department of Hematology, Dupuytren Hospital University Center of Limoges, Limoges Cedex, France; 5 Department of Internal Medicine, Dupuytren Hospital University Center of Limoges, Limoges Cedex, France; 6 Department of Immunology, Dupuytren Hospital University Center of Limoges, Limoges Cedex, France

**Keywords:** Chronic lymphocytic leukaemia, Cell signalling

## Abstract

Evading apoptosis and sustained survival signaling pathways are two central hallmarks of B-cell chronic lymphocytic leukemia (B-CLL) cells. In this regard, nurse-like cells (NLC), the monocyte-derived type 2 macrophages, deliver stimulatory signals via B-cell activating factor (BAFF), a proliferation-inducing ligand (APRIL), and the C-X-C Motif Chemokine Ligand 12 (CXCL12). Previously, we demonstrated that brain-derived neurotrophic factor (BDNF) protects B-CLL cells from spontaneous apoptosis by activating the oncogenic complex NTSR2-TrkB (neurotensin receptor 2-tropomyosin-related kinase receptor B), only overexpressed in B-CLL cells, inducing anti-apoptotic protein Bcl-2 (B-cell lymphoma 2) expression and Src kinase survival signaling pathways. Herein, we demonstrate that BDNF belongs to the NLC secretome and promotes B-CLL survival. This was demonstrated in primary B-CLL co-cultured with their autologous NLC, compared to B-CLL cells cultured alone. Inhibition of BDNF in co-cultures, enhances B-CLL apoptosis, whereas its exogenous recombinant activates pro-survival pathways in B-CLL cultured alone (i.e. Src activation and Bcl-2 expression), at a higher level than those obtained by the exogenous recombinant cytokines BAFF, APRIL and CXCL12, the known pro-survival cytokines secreted by NLC. Together, these results showed that BDNF release from NLC trigger B-CLL survival. Blocking BDNF would support research strategies against pro-survival cytokines to limit sustained B-CLL cell survival.

## Introduction

B lymphocytes from patients with chronic lymphocytic leukemia (B-CLL) are resistant to apoptosis^1,2^ and accumulate in lymphoid tissues in which the microenvironment provides long-term protection and allows cancer progression^3^. Indeed, nurse-like cells (NLC), which are type II macrophages differentiated in the presence of B-CLL cells^4^, act as tumor-associated macrophages (TAMs)^5^ by releasing pro-survival cytokines such as B-cell activating factor (BAFF), a proliferation-inducing ligand (APRIL), and stromal cell-derived factor 1 (SDF-1) [also called CXCL12 (C-X-C Motif Chemokine Ligand 12)]^4,6,7^. Together, these soluble factors build a microenvironment within the lymph nodes and bone marrow that promotes homing and survival of B-CLL cells^3^. Moreover, this mechanism might contribute to maintain therapy-selected subclones survival associated with proliferation and poor patient outcome^8,9^.

Indeed, isolated B-CLL cells die, illustrating the importance of the microenvironment in vivo^4,7,10^. Strikingly, long-term culture of peripheral blood mononuclear cells (PBMCs) with B-CLL cells triggers differentiation of monocytes into NLC, which maintain survival signaling pathways in remaining B-CLL cells^3,11^. These in vitro NLC mimic the pro-survival centers observed in lymph nodes, which deliver stimulatory signals. Interestingly, while inhibiting BAFF, APRIL, and CXCL12 limits B-CLL survival, some B-CLL cells remain alive^4,7,10^, suggesting that unknown factors in the NLC microenvironment are essential for evasion of apoptosis.

We previously demonstrated that B-CLL survival under such conditions depends on signaling via the oncogenic complex NTSR2-TrkB expressed at the B-CLL cell surface^12^. Indeed, NTSR2, a G protein-coupled receptor (GPCR), is overexpressed in CLL cells, while neurotensin (NTS), its natural ligand, is present at markedly lower concentrations in serum from CLL patients. However, interaction between NTSR2 and the tyrosine kinase receptor tropomyosin-related kinase B (TrkB) forms a conditional oncogene complex in the presence of the TrkB ligand brain-derived neurotrophic factor (BDNF). NTSR2-TrkB-BDNF pro-survival signaling is marked via the overexpression of the anti-apoptotic protein Bcl-2 as well as by inducing Src kinase-mediated survival signaling pathways^12^. Inversely, NTSR2 downregulation or TrkB inhibition triggers massive apoptosis^12^. Because survival centers formed by NLC protect B-CLL cells from spontaneous apoptosis, and since NTSR2-TrkB promotes survival signals, it is tempting to speculate that BDNF is part of the NLC microenvironment and plays a crucial role in apoptosis evasion.

Here, we demonstrate that BDNF belongs to the NLC secretome and protects B-CLL cells from apoptosis. Indeed, co-culture models incorporating an anti-BDNF blocking antibody reveal the crucial and direct role played by BDNF during Src phosphorylation and Bcl-2 expression in B-CLL cells. Here, we propose a model in which BDNF or pro-survival cytokines secreted by the NLC within survival centers act together to promote survival of B-CLL cells.

## Results

### NLC mediate NTSR2-TrkB expression in B-CLL

**Figure 1:**
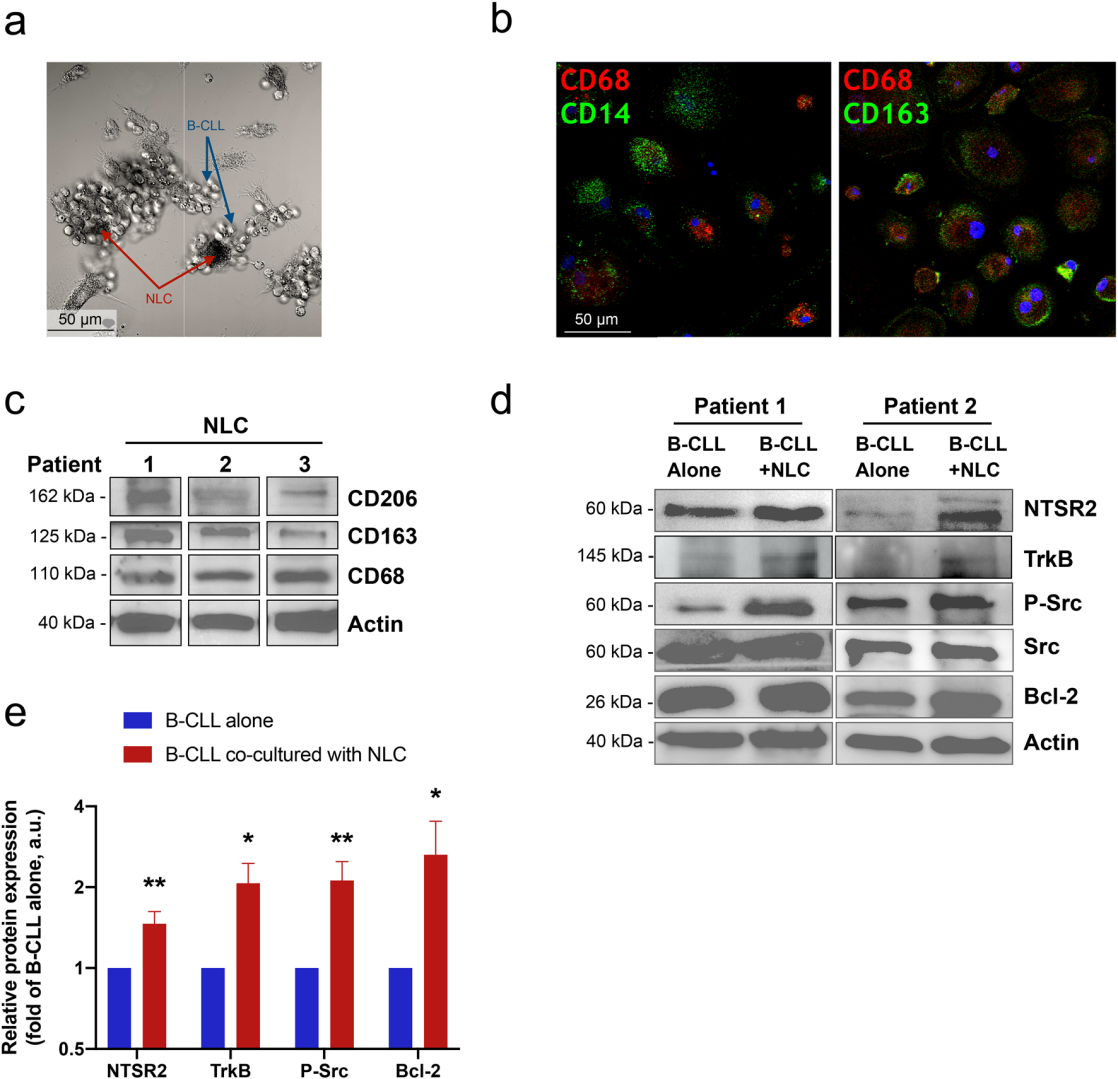
NLC drive expression of NTSR2-TrkB and pro-survival signaling in B-CLL cells. (**a**) Brightfield microscopy of co-cultured NLC (red arrows) and B-CLL cells (blue arrows). (**b**) Confocal microscopy analysis of CD14 (green) and CD68 (red) (left panel), and CD163 (green) and CD68 (red) (right panel), expression by NLC (n = 3). Nuclei are stained with DAPI. (**c**) Western blot analysis of CD68, CD163, and CD206 expression by NLC (n = 3). (**d**) Representative western blots showing expression of NTSR2, TrkB, p-Src, Src, and Bcl-2 by B-CLL cells isolated from two independent patients and cultured alone or with autologous NLC for 48 h. (**e**) Quantification of NTSR2 (n = 15), TrkB (n = 6), p-Src/Src (n = 17) and Bcl2 (n = 16) expression in independent patient samples. Data are presented as the mean ± SEM from at least three independent experiments (*p < 0.05 and **p < 0.01). Blots are cropped for clarity; full-length blots are shown in the Supplementary Fig. S7.

Based on our first report examining the role of the conditional oncogenic complex NTSR2-TrkB, expressed on B-CLL (Supplementary Fig. S1) in maintaining their survival, we speculated that NLC increase expression of this complex in pro-survival centers. To investigate this hypothesis further, we generated a NLC model based on the protocol of Burger et al.^4^ by setting up long-term cultures of PBMCs isolated from CLL patients. Indeed, after 2 weeks, surviving B-CLL lymphocytes remained agglutinated to large adherent cells exhibiting morphological features characteristic of NLC (Fig. 1a). To confirm that these culture conditions generated NLC cells, we performed immunostaining for NLC markers after removing floating CLL cells that comprised 90% of cultured cells. As expected, adherent cells co-expressed CD14, CD68, and CD163 providing evidence of monocyte differentiation into NLC (Fig. 1b). Likewise, NLC expressed BAFF, APRIL (Supplementary Fig. S2). Moreover, lysates prepared from adherent cells and analyzed by western blotting revealed expression of NLC markers CD68, CD163, and CD206 (Fig. 1c).

Next, we cultured NLC with autologous B-CLL cells for 48 h to explore whether the microenvironment maintained survival pathways and activated of B-CLL cells. Indeed, co-culture led to a marked increase in both Src phosphorylation and expression of the anti-apoptotic protein Bcl-2, suggesting that NLC provide a pro-survival microenvironment for B-CLL cells (Fig. 1d and e). Interestingly, we also observed that co-culture triggered expression of both NTSR2 and TrkB by B-CLL cells (Fig. 1d and e), highlighting that NLC induce survival pathways through this conditional oncogenic complex.

Taken together, these results suggest that NLC modulate expression of NTSR2-TrkB at the B-CLL cell surface; therefore, we hypothesized that BDNF, the ligand for this conditional complex, may belong to the NLC secretome.

### BDNF belongs to the NLC secretome

**Figure 2:**
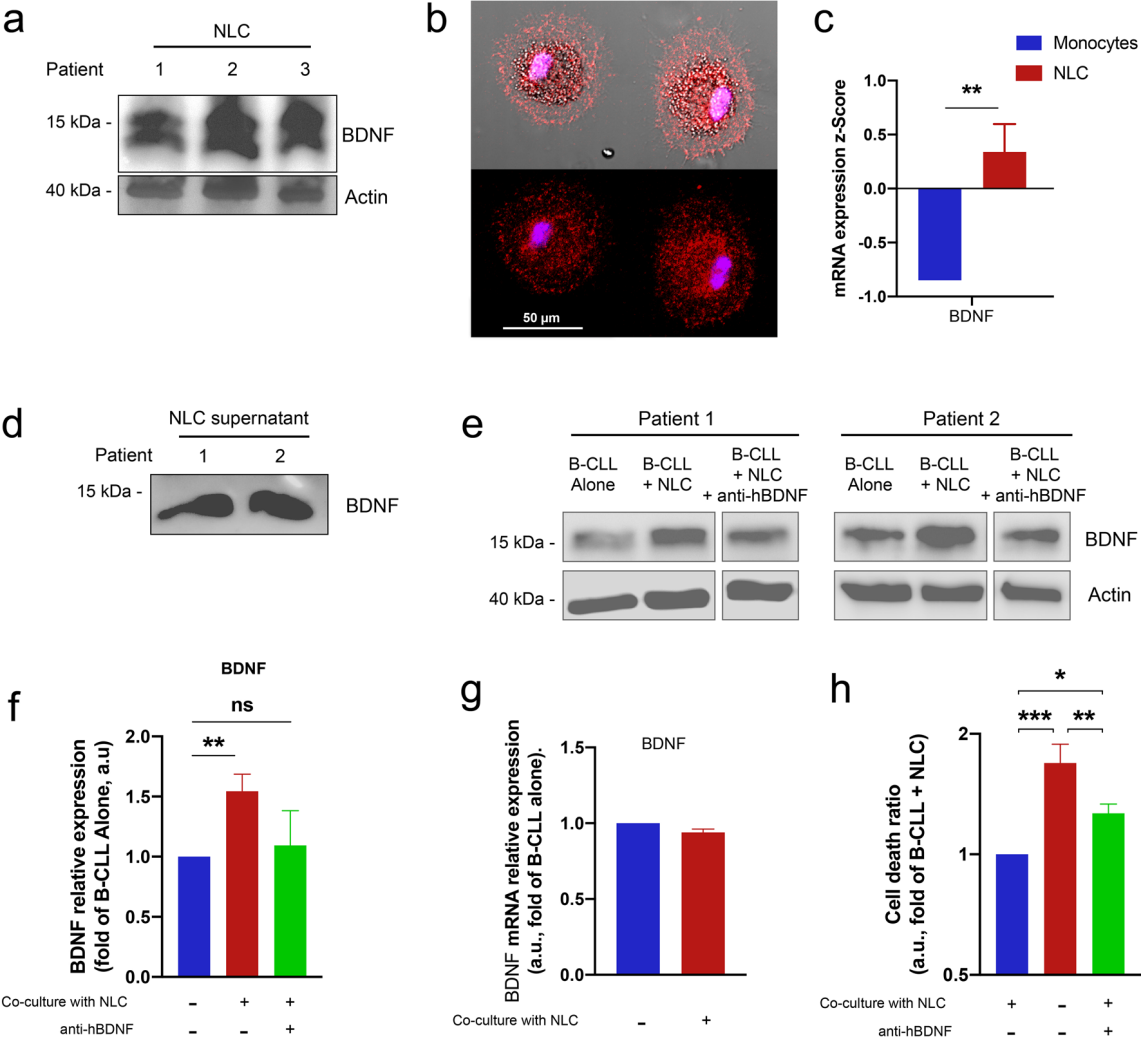
NLC secrete BDNF, thereby protecting B-CLL cells from apoptosis. (**a**) Representative western blots showing expression of BDNF in lysates of NLC from two independent patient samples (n = 5). (**b**) Immunofluorescence analysis of BDNF (red) expression in NLC by confocal microscopy. (**c**) Relative expression of *BDNF* mRNA by normal healthy monocytes (n = 6) and NLC isolated from B-CLL patients (n = 6), as determined by RT-qPCR. (**d**) Representative western blots showing BDNF expression in supernatants of two independent NLC culture (n = 6). (**e**) Representative western blots showing BDNF expression by B-CLL cells cultured alone, with autologous NLC, or with autologous NLC plus an anti-BDNF blocking antibody (anti-hBDNF; 200 ng/mL) for 48 h. The three conditions for each patient assessed on the same western blot, which has been cropped to present only relevant data. The uncropped western blot membranes are shown in Supplementary Fig. S4 (**f**) Quantification of BDNF protein from independent patient samples (n = 5). (**g**) Relative expression of *BDNF* mRNA by B-CLL cells cultured alone or with autologous NLC for 48 h, as determined by RT-qPCR (n = 7). (**h**) Flow cytometry analysis of cell death, as assessed by annexin V-fluorescein isothiocyanate/propidium iodide dual staining of B-CLL cells cultured for 72 h either alone, with autologous NLC, or with autologous NLC plus an anti-BDNF antibody (200 ng/mL). Cell death was assessed by excluding annexin V/propidium iodide-negative cells. Experiments were performed using n = 9 patient samples. Data are presented as the mean ± SEM from at least three independent experiments (*p < 0.05, **p < 0.01, ***p < 0.001). *ns* not significant. Blots are cropped for clarity; full-length blots are shown in the Supplementary Fig. S7.

To investigate whether NLC secrete BDNF, we performed immunofluorescence and western blot analysis of NLC lysates and their respective cell culture supernatants. When floating B-CLL cells were removed from the cultures, we observed that BDNF remained expressed (Fig. 2a, b) and secreted (Fig. 2d) by resting adherent cells in culture corresponding solely to NLC as detected by their respective markers CD14, CD68 and CD163 in culture (Fig. 1b). These results demonstrate for the first time that BDNF is part of the NLC microenvironment. Likewise, transcriptional analysis revealed that expression of BDNF increased significantly (*p* = 0.0042) upon differentiation of CLL monocytes into NLC (Fig. 2c). Surprisingly, expression of BDNF in B-CLL cell lysates increased markedly upon co-culture with NLC (Fig. 2e, f); however, expression of *BDNF* mRNA was the same as that by B-CLL cells cultured alone (Fig. 2g and Supplementary Fig. S3). These results might suggest that BDNF is provided by NLC. Indeed, neutralization of BDNF using a monoclonal antibody (anti-hBDNF) reduced the amount of BDNF in B-CLL cell lysates (Fig. 2e, f), suggesting that BDNF is part of the communication network between NLC and B-CLL cells. Taken together, these data strengthen the hypothesis that NLC are a source of BDNF, which once fixed at the B-CLL cell surface by the conditional complex NTSR2–TrkB, enable evasion of apoptosis (Fig. 2h). Indeed, evaluation of B-CLL cell death using annexin V/propidium iodide double staining revealed that while the NLC microenvironment promotes B-CLL cell survival, the survival benefits are limited in the absence of BDNF (Fig. 2h). Thus, BDNF participates actively in NLC–B-CLL crosstalk and assumes a crucial role in enabling B-CLL cells to evade apoptosis.

### BDNF activates NTSR2 expression and pro-survival signals in B-CLL to the same extent as BAFF, APRIL, and CXCL12 combined

**Figure 3:**
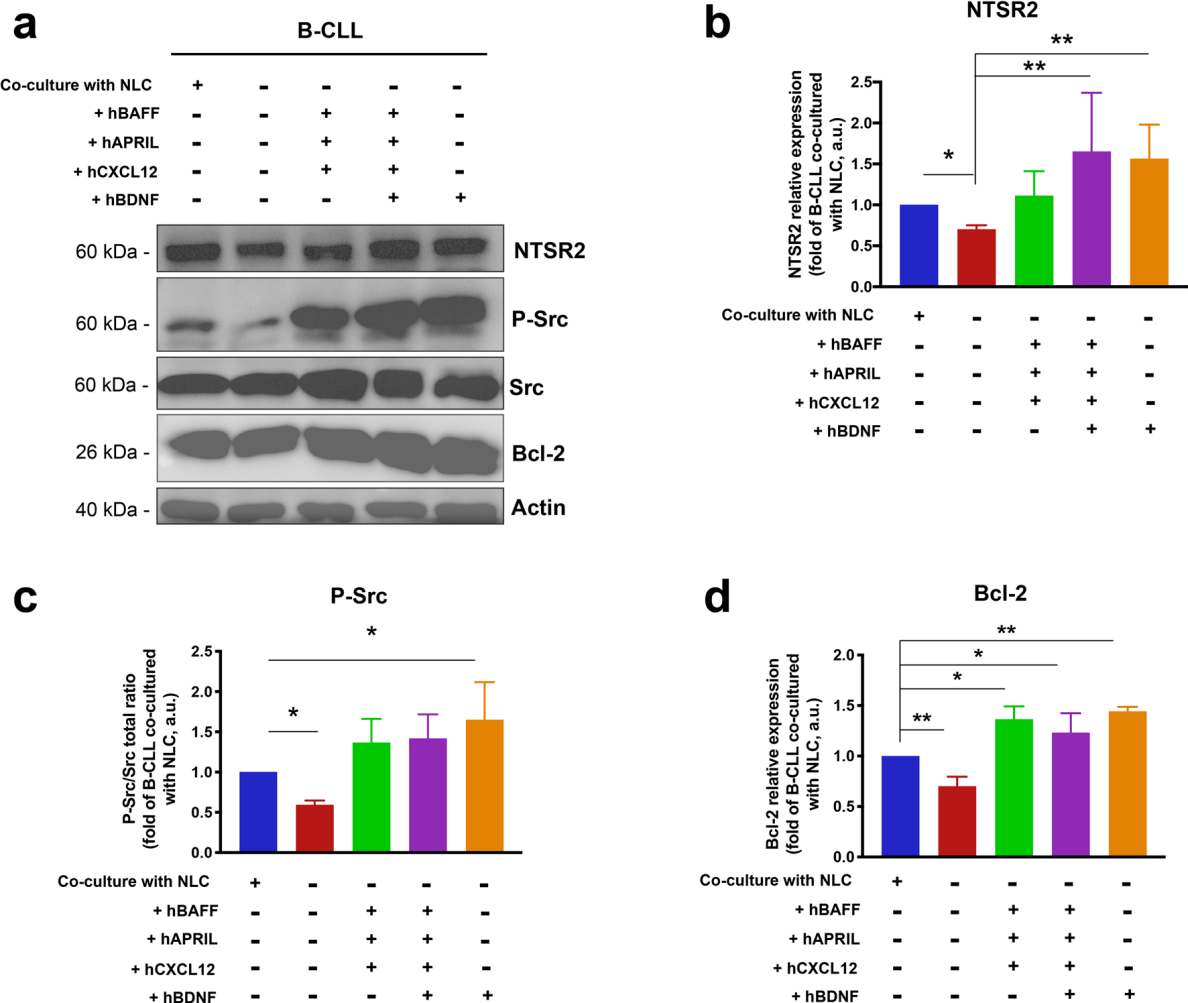
The effects of BDNF on B-CLL pro-survival signaling are similar to those of BAFF, APRIL, and CXCL12 combined. (**a**) Representative western blot showing expression of NTSR2, p-Src, Src, and Bcl-2 by B-CLL cells isolated from patients. Cells were either co-cultured for 48 h with autologous NLC or cultured alone in complete medium supplemented (as indicated) with exogenous human (h) CXCL12 (100 ng/mL), BAFF (2 ng/mL), APRIL (25 ng/mL), or BDNF (100 ng/mL). (b,c,d), full-length blots are shown in the Supplementary Fig. S7. Quantification of NTSR2 (**b**), p-Src (**c**), and Bcl-2 (**d**) expression in four different patient samples. Data are presented as the mean ± SEM from at least three independent experiments, (*p < 0.05, **p < 0.01). Blots are cropped for clarity; full blots are shown in the Supplementary Fig. S7.

To further delineate the role of BDNF in the NLC microenvironment, we artificially generated an NLC secretome by combining human cytokines BAFF (+hBAFF), APRIL (+hAPRIL), and CXCL12 (+hCXCL12) and applied them to isolated B-CLL cells. Surprisingly, while this cytokine combination strongly mimicked co-culture conditions i.e., increasing NTSR2 expression (Fig. 3a, b) and triggering Src phosphorylation and Bcl-2 expression (Fig. 3a, c, d), there was no additive effect after inclusion of BDNF. These results suggest that BDNF plays a minor role when combined with other pro-survival cytokines. However, when used alone, BDNF increased NTSR2 expression (Fig. 3a, b) and triggered levels of Src phosphorylation (Fig. 3c) and Bcl-2 expression (Fig. 3d) similar to those triggered by the pro-survival cytokine combination. Thus, the protective role of BDNF alone is equivalent to that of a combination of pro-survival cytokines, suggesting that BDNF may have independent effects on B-CLL survival.

### Blocking BDNF is crucial to reduce NTSR2 expression and enhance APRIL, BAFF, CXCL12 inhibition

**Figure 4:**
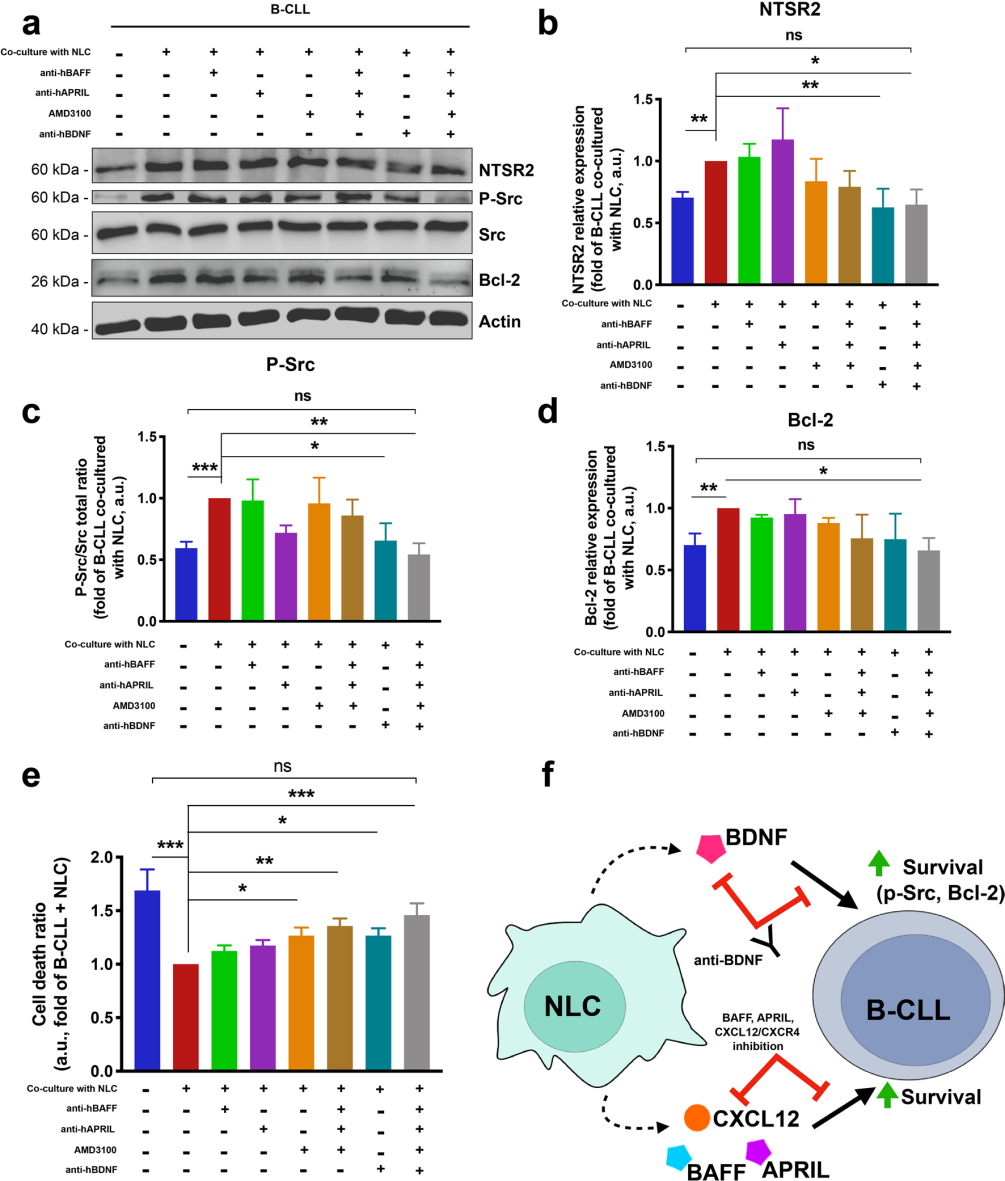
Inhibiting BDNF in addition to BAFF, APRIL, and CXCR4 reverses NLC-mediated protection of B-CLL cells from apoptosis. (**a**) Representative western blot showing expression of p-Src and Bcl-2 by B-CLL cells isolated from patients. Cells were cultured (for 72 h) alone, with autologous NLC, or with autologous NLC plus single or combined inhibition of BAFF (anti-hBAFF, 100 ng/mL), APRIL (anti-hAPRIL, 500 ng/mL), CXCL12 receptor CXCR4 (AMD3100, 0.5 µg/mL), and BDNF (anti-hBDNF, 200 ng/mL). (**b**–**d**), full-length blots are shown in the Supplementary Fig. S7.Quantification of NTSR2 (**b**), p-Src (**c**) and Bcl-2 (**d**) expression in six independent experiments using six different patient samples. (**e**) Flow cytometry analysis of cell death, assessed by annexin V-fluorescein isothiocyanate/propidium iodide dual staining of B-CLL cells cultured (for 72 h) either alone, with autologous NLC, or with autologous NLC and single or combined inhibition of BAFF (anti-hBAFF, 100 ng/mL), APRIL (anti-hAPRIL, 500 ng/mL), CXCL12 receptor CXCR4 inhibition (AMD3100, 0.5 µg/mL), and BDNF (anti-hBDNF, 200 ng/mL). Cell death was assessed by exclusion of annexin V/propidium iodide-negative cells. Experiments were performed using n = 9 different patient samples. Data are presented as the mean ± SEM from at least three independent experiments (*p < 0.05, **p < 0.01, ***p < 0.001). *ns* not significant. (**f**) Schematic representation of the obtained results. NLC produce and secrete BDNF, which promotes survival of B-CLL cells by activating the Src signaling pathway and upregulating expression of Bcl-2. This newly described member of the NLC secretome appears to exert both complementary (alongside BAFF, APRIL and CXCL12) and independent effects. Here, we propose a model in which BDNF or pro-survival cytokines secreted by NLC within survival centers balance each other out to facilitate survival of B-CLL cells, and argue that simultaneous inhibition of BDNF signaling through the NTSR2-TrkB conditional oncogenic platform, along with inhibition of BAFF, APRIL and CXCR4/CXCL12, could cancel out NLC-mediated protection of B-CLL cells from apoptosis and restore normal cell death.

Next, to assess whether BDNF alone allows B-CLL cells to increase NTSR2 and to evade from apoptosis, we co-cultured B-CLL cells with NLC and inhibited each pro-survival cytokine [(using anti-BAFF, -APRIL, and CXCL12 receptor CXCR4 (C-X-C chemokine receptor type 4) antagonist AMD3100], in the presence/absence of an anti-BDNF antibody. In co-culture with NLC, the inhibition of BDNF alone or in combination with the co-inhibition of BAFF, APRIL and CXCR4 decreased NTSR2 expression of B-CLL to a level similar to that was obtained in B-CLL alone (Fig. 4a, b). Then, we assessed survival by measuring Src phosphorylation and Bcl-2 expression before evaluating B-CLL cell death by annexin V/propidium iodide double staining. Interestingly, Src phosphorylation remained unchanged upon either individual or co-inhibition of BAFF, APRIL, and CXCR4; phosphorylation levels changed only after neutralization of BDNF (Fig. 4a, c). Surprisingly, co-inhibition of pro-survival cytokines or neutralization of BDNF alone did not decrease expression of Bcl-2, suggesting that some sort of cooperation between pro-survival cytokines and BDNF with respect to maintenance of B-CLL survival (Fig. 4a, d). Indeed, to reduce Src phosphorylation and Bcl-2 expression to levels observed in B-CLL monocultures, co-cultures required neutralization of BDNF plus co-inhibition of BAFF, APRIL, and CXCR4 (Fig. 4a, c and d).

Loss of the protective function provided by the NLC microenvironment was evidenced by increased cell death at 72 h when B-CLL cells were cultured alone or when BDNF and pro-survival cytokines were inhibited in co-cultures. Likewise, the finding that inhibition of individual cytokines or BDNF in co-cultures did not abrogate the protective properties of NLC on B-CLL cells (Fig. 4e) highlighted the co-operative effects. Overall, these results shed light on cooperation between CXCL12, BAFF, APRIL, and BDNF during communication between NLC and B-CLL cells. In the presence of NLC, the NTSR2-TrkB-BDNF axis in B-CLL cells is upregulated, resulting in increased pro-survival signaling through the Src pathway and upregulation of Bcl-2. Only combined inhibition of CXCR4, BAFF, APRIL, and BDNF fully abolished NLC-mediated protection of B-CLL cells (Fig. 4f).

## Discussion

B-CLL lymphocytes are resistant to apoptosis and accumulate in lymphoid organs and peripheral blood^1,2^. Evasion of apoptosis is maintained by stimulation of pro-survival signaling pathways^13,14^ and high expression of the anti-apoptotic protein Bcl-2^15^. These hallmarks of B-CLL appear related to B-cell receptor (BCR) activation and subsequent downstream signals. Clinical trials targeting the BCR limit both progression of CLL and the disease course. However, the clinical benefits inevitably decline, highlighting the existence of bypass mechanisms. In this regard, the microenvironment surrounding B-cell malignancies has attracted much attention. Indeed, NLC play an important role in the physio-pathological feature of CLL by providing a favorable environment. Indeed, NLC promote recruitment, survival, and proliferation of B-CLL cells^11^ by secreting soluble mediators^4,7^. They produce large quantities of CXCL12^4^, which is implicated in both chemotaxis and survival of B-CLL cells through activation of the ERK and Akt signaling pathways^4,7^. BAFF and APRIL are also secreted by NLC^16^; these cytokines promote survival by inducing expression of Bcl-2^7^. However, BAFF, APRIL, and CXCL12 only provide partial protection from apoptosis when compared with NLC^4,7^. This NLC-mediated protection from apoptosis suggests that disrupting crosstalk between B-CLL cells and NLC is crucial to limiting both formation of pro-survival centers and treatment relapse.

In this regard, we previously identified a pro-survival signaling pathway in circulating B-CLL cells and implicated a complex formed by NTSR2 and TrkB^12^. Similar to other known GPCR-TKR signaling platforms^17,18^, NTSR2 is transactivated by TrkB upon binding of BDNF. This complex engages survival signals via the Src, MAPK, and PI3K/Akt pathways, leading to expression of anti-apoptotic Bcl-2 family proteins^12^.

Interestingly, we found herein that co-culture of B-CLL cells with NLC increased expression of NTSR2 and TrkB by B-CLL cells, as well as inducing Src pro-survival signals and expression of Bcl-2. Hence, we hypothesized that BDNF belongs to the NLC secretome. Indeed, we found that BDNF was secreted by NLC and activated survival pathways in B-CLL cells. BDNF secreted by NLC stimulated NTSR2 expression and activation of the conditional complex NTSR2-TrkB, the formation of which is stimulated by BDNF^12^, as demonstrated by the decrease in NTSR2 expression and induction of apoptosis following BDNF neutralization by a blocking antibody. We chose to inhibit BDNF using a blocking antibody rather than by siRNA-mediated inhibition in NLC generated from patient samples because transfection efficiency and reproducibility were extremely low.

Stimulation of isolated B-CLL cells with BDNF alone activated Src signaling and upregulated Bcl-2, as observed in NLC co-cultures or after exposure to BAFF, APRIL, and CXCL12. These results highlight the high potency of BDNF to trigger pro-survival signals in B-CLL cells. However, BDNF stimulation in the presence of pro-survival cytokines did not increase Src phosphorylation and Bcl-2 expression, suggesting that (at least in vitro) these proteins might have a redundant effect and may have saturated the pro-survival signaling pathway. Interestingly, inhibiting BDNF alone reduced Src phosphorylation to a greater extent than combined inhibition of BAFF, APRIL, and CXCR4. Strikingly, expression of Bcl-2 remained unchanged upon neutralization of BDNF or inhibition of pro-survival cytokines, suggesting existence of a compensatory mechanism. Indeed, this hypothesis was supported by the observation that both Src phosphorylation and Bcl-2 expression fell only after co-inhibition of all four soluble mediators (BAFF, APRIL, CXCR4, and BDNF). Targeting all four soluble mediators abolished NLC-mediated Src signaling and Bcl-2 expression completely, resulting in levels of cell death close to those observed when B-CLL cells were cultured alone. Taken together, the results suggest that the NLC secretome mediates apoptosis resistance through a combination of soluble mediators: CXCL12, BAFF, APRIL, and BDNF.

Over the past two decades, the CLL microenvironment has emerged as a key regulator of B-CLL cell survival and proliferation^3,19^. Here, we demonstrate that NLCs promote pathways of resistance to apoptosis in vitro via secretion of BDNF and hypothesize that the same pathway could operate in lymph node proliferation centers (in which cells reside in vivo) although there is still no experimental data to support this.

NTSR2-TrkB-BDNF is overexpressed by circulating B-CLL cells and plays an important role in apoptosis resistance^12^; its expression is upregulated further by the microenvironment provided by NLC. Therefore, we propose that targeting and inhibiting this pathway will have beneficial therapeutic outcomes.

## Materials and methods

### Primary cultures

Forty patients with B-CLL (clinical data provided in Supplementary Table 1) were included in the study, which was approved by the Institutional Review Board (AC 72-2011-18). NLC were obtained as described previously^4^. Briefly, PBMCs were isolated by Ficoll gradient centrifugation (Eurobio, Les Ulis, France), 25 min at 700 g. PBMCs were seeded at 10^7^ cells/mL in RPMI-1640 medium completed with 10% Fetal Calf Serum (IDBio, France), 1% Non-Essential Amino Acids (Gibco), 1% MEM Vitamins (Gibco) and 1% Penicillin/Streptomycin (Gibco). After 14 days of culture in an incubator at 37 °C with 5% CO_2_, cells were separated in two populations, adherent NLC and the remaining floating cells. Floating cells are harvested then adherent cells are thoroughly washed with PBS until only NLC remain visible by microscopy and confirmed by immunofluorescence with NLC markers (CD14, CD68 and CD163). At this point, NLC were maintained to co-culture experiments (48 h) or harvested by trypsin for protein analyses. B-CLL cells are immuno-selected from PBMCs using the B-CLL Cell Isolation Kit, human (Miltenyi Biotec, Bergisch Gladbach, Germany), according to the manufacturer’s instructions. Whatever conditions, B cell purity was higher than 95% (Supplementary Fig. S5). For co-culture experiment, purified B-CLL are either put back in co-culture with autologous NLC or cultured alone in completed RPMI-1640, with or without additional treatment (48 h). Culture protocol flow-through is summarized in Supplementary Fig. S6.

### Treatments

Cultured cells were incubated with 100 ng/mL human recombinant CXCL12^20^ (BioLegend, San Diego, CA, USA), 2 ng/mL human recombinant BAFF (BioLegend), and 25 ng/mL human recombinant APRIL (Peprotech, Neuilly-Sur-Seine, France) (these concentrations are comparable with the respective physiological levels in plasma from CLL patients^21,22^) or 100 ng/mL human recombinant BDNF (Alomone Labs, Jerusalem, Israel), as described previously^12,23^. BAFF was neutralized using a goat anti-BAFF polyclonal antibody (20 ng/mL; #AF124, R&D Systems, Minneapolis, MN, USA) and APRIL was neutralized using a mouse anti-APRIL monoclonal antibody (1 μg/mL; #MAB5860, R&D Systems)^10^. CXCR4 was inhibited using AMD3100 (0.5 μg/ml; Merck Millipore, Fontenay sous Bois, France) to reduce the CXCR4-positive cell population markedly^24^. BDNF was neutralized using an anti-BDNF mouse monoclonal antibody (200 ng/mL; #GF35L, Merck Millipore).

### Immunofluorescence analysis

Isolated B-CLL cells or PBMCs were from B-CLL patients were cultured at high density (10 million per mL) in 24-well plates containing glass coverslips (14 mm diameter, Menzel-Gläser, VWR) to obtain NLC. Immunofluorescence staining was performed as described previously^12^ using the following primary antibodies: mouse monoclonal anti-CD14-Alexa488 (1:20, #325610, BioLegend, San Diego, CA, USA), anti-CD68 (1:20, #333801, BioLegend), and mouse monoclonal anti-CD163 (1:20, #326502, BioLegend), anti-BAFF (1:500, #orb76960, Biorbyt, Cambridge, UK), anti-APRIL (1 µg/mL; #AF884, R&D Systems). Slides were observed under a confocal microscope (LSM 880, Carl Zeiss, Oberkochen, Germany) and data were analyzed using Zeiss ZEN software.

### Western blot analysis

Proteins were extracted as described previously^25^. Western blotting was carried out using 40 μg aliquots of protein extract, as previously described^12^. The following primary antibodies were used: rabbit polyclonal anti-NTSR2 (1:400, #ANT-016, Alomone Labs), mouse monoclonal anti-BDNF (1:1,000, #MAB648, R&D Systems), rabbit polyclonal anti-TrkB (1:500, #orb214339, Biorbyt), mouse monoclonal anti-CD68 (1:500, #333801, BioLegend), mouse monoclonal anti-CD163 (1:500, #326502, BioLegend), mouse monoclonal anti-CD206 (1:500, #32502, BioLegend), rabbit polyclonal anti-phospho-Src Tyr-416 (1:1,000, #2,101, Cell Signaling Technology, Ozyme, France), rabbit polyclonal anti-Src (1:1,000, #2,108, Cell Signaling Technology, Ozyme), rabbit monoclonal anti-Bcl-2 (1:1,000, #2,870, Cell Signaling Technology, Ozyme), anti-BAFF (1:1,000, #orb76960, Biorbyt), anti-APRIL (0.2 µg/mL; #AF884, R&D Systems) and mouse monoclonal anti-β-actin (1:10,000, #A5441, Sigma-Aldrich). Protein expression was quantified using ImageJ software (NIH). Full-length Western Blot membranes are provided in the Supplementary Data. Because primary cultures provided low protein yield, several patient’s samples could only be analyzed once. Hence, by cutting membranes we optimized data collection and analysis of several proteins at different sizes without repeating membrane stripping. As a result, whole membranes are not available for all experiments, but the full-length of truncated membranes are provided in Supplementary Informations.

### Real-time quantitative PCR

RNA extraction, reverse transcription, and real-time quantitative PCR were performed as described previously^26^ using a QuantStudio™ 5 Real-Time PCR System (Thermo Fisher Scientific). Primers and probes targeting reference genes were obtained from Thermo Fisher Scientific TaqMan® Gene Expression Assays; Hs01060665_g1 and Hs99999901_s1, were used to target *ACTB* and *18S*, respectively. The following TaqMan® primers and probe were designed to target BDNF: forward, 5′-GGCTATGTGGAGTTGGCATT-3′, and reverse 5′-CAAAACGAAGGCCTCTGAAG-3′; probe, 5′-ATTTCTGAGTGGCCATCCCAAGGTCTAG-3′. Relative mRNA levels were determined after normalization to both *18S* and *ACTB*.

### Cell death analysis

Cell death was assessed using a previously described propidium iodide/annexin-V-FITC double staining protocol^27^. Experiments were conducted using nine different patient samples per condition. Analyses were performed on Fortessa flow cytometer (Becton Dickinson, France) and results were analyzed using Flowlogic software (Miltenyi Biotec).

### Microarray data mining

Transcriptome data obtained using the Affymetrix Human Genome U133 Plus 2.0 platform were downloaded from the NCBI repository Gene Expression Omnibus (GEO) database; the dataset accession number is GSE13811^28^ including B-CLL cells alone (n = 9) or B-CLL cells co-cultured with NLC (n = 9). The *BDNF* probe ID was 239367_at.

### Data treatment and statistical analysis

Graphical representations were generated using GraphPad Prism 7 software. Immunohistochemistry and western blot analysis were quantified using ImageJ software (NIH). Results were analyzed by one-way ANOVA, followed by Fisher’s post-hoc test. Analyses were performed using StatView 5.0 software (Abacus Concepts, Piscataway, NJ, USA). A *p* value < 0.05 was considered significant. Data were obtained from at least three independent experiments and are presented as the mean and standard error of the mean (SEM).

### Ethical approval and consent to participate

The study was approved by the Limoges University Hospital Institutional Review Board, AC 72-2011-18. All patients provided informed consent to participate, all experiments were performed in accordance with relevant named guidelines and regulation.

## Supplementary information

Supplementary Information.

## Data Availability

The dataset analyzed during the current study is available from the GEO repository. https://www.ncbi.nlm.nih.gov/geo/query/acc.cgi?acc=GSE13811.
