# Supplementary material for: BDNF belongs to the nurse-like cell secretome and supports survival of B chronic lymphocytic leukemia cells
Source: Sci Rep. 2020 Jul 28;10:12572. doi: 10.1038/s41598-020-69307-1 (PMC7387561; doi:10.1038/s41598-020-69307-1)
Supplement: Supplementary file 1 — Supplementary Information. [file 41598_2020_69307_MOESM1_ESM.docx]

**Supplementary Informations**

**BDNF belongs to the nurse-like cell secretome and supports survival of B chronic lymphocytic leukemia cells**

Hugo Talbot, Sofiane Saada, Elodie Barthout, Paul-François Gallet, Nathalie Gachard, Julie Abraham, Arnaud Jaccard, Danielle Troutaud, Fabrice Lalloué, Thomas Naves, Anne-Laure Fauchais and Marie-Odile Jauberteau

**Supplementary Table 1: Clinical characteristics of CLL patients’ cohort**


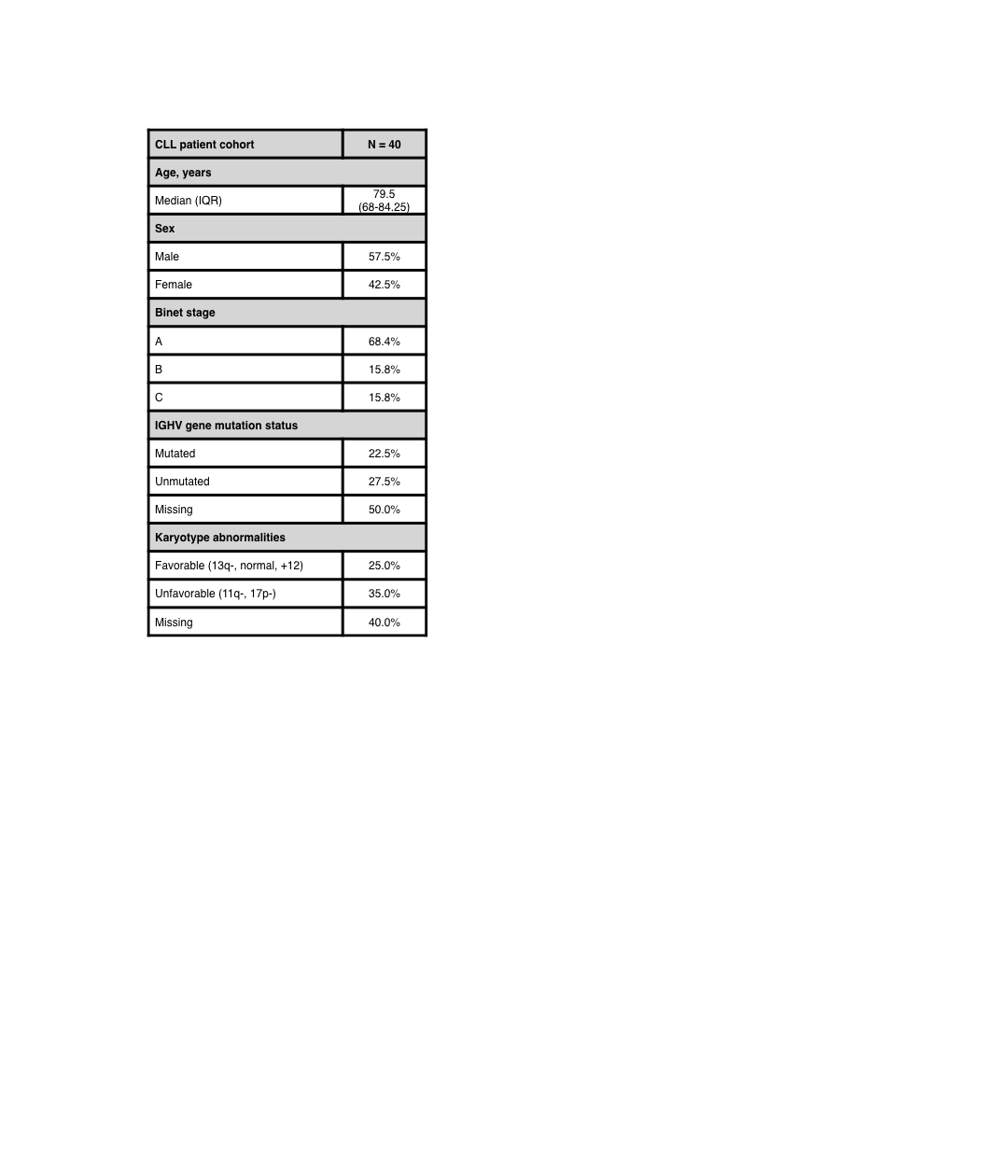


**Supplementary Figure S1:**

**
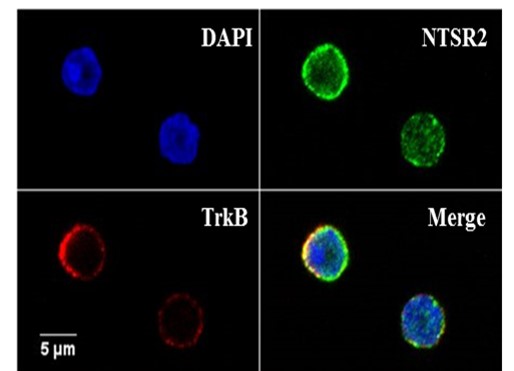
**

***NTSR2 and TrkB expression in purified B-CLL***

Confocal microscopy analysis of NTSR2 (green) and TrkB (red) in B-CLL and their co-localization (yellow staining, in the merge image) in B cells from CLL Patient. Nuclei were stained by using DAPI.

**Supplementary Figure S2:**

**
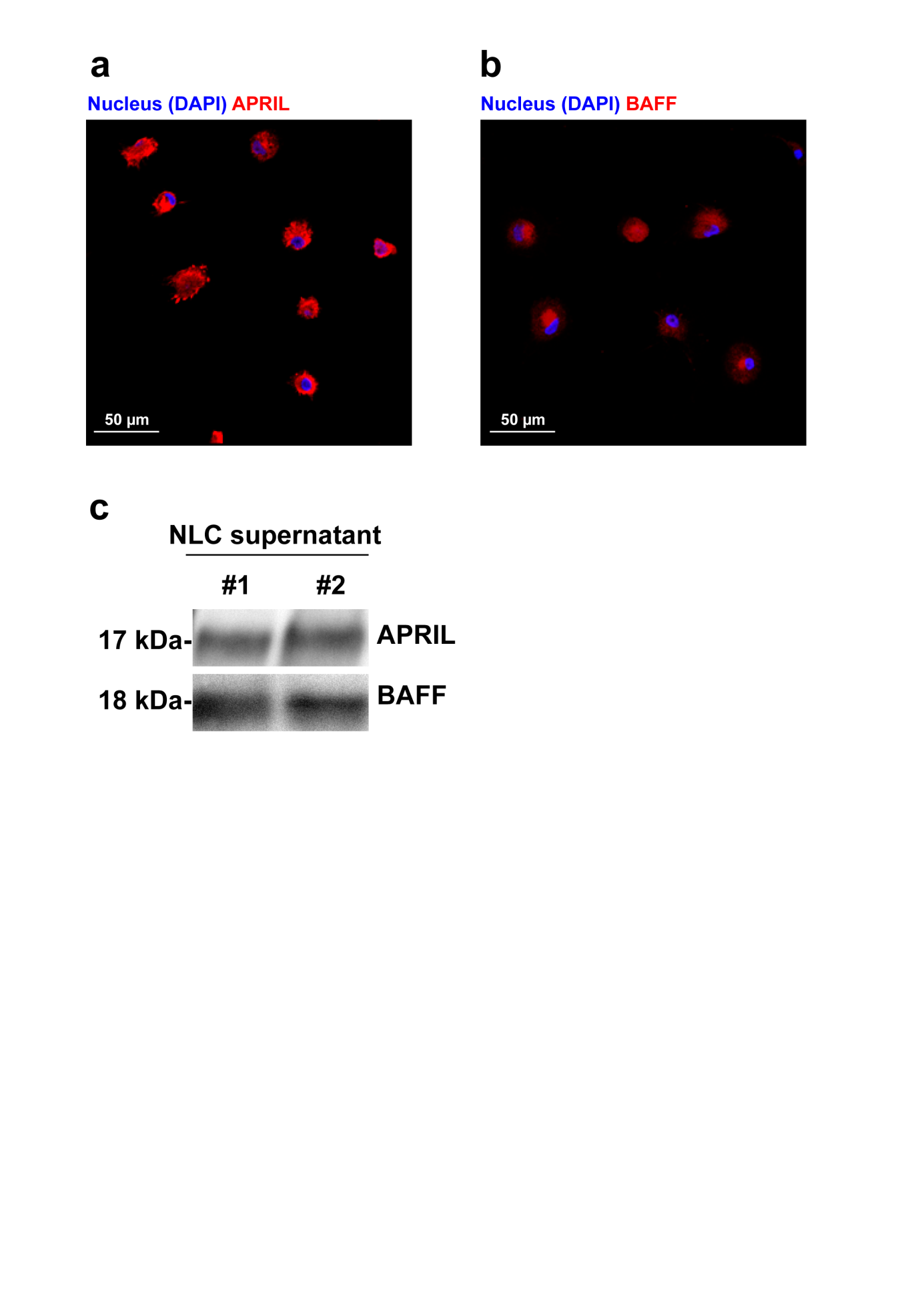
**

***APRIL and BAFF expression in Nurse Like Cells***

(a) Confocal microscopy analysis of APRIL (red) and (b) BAFF (red) by NLC. Nuclei are stained with DAPI. (c) Representative western blots showing APRIL and BAFF expression in two independent NLC culture supernatants.

**Supplementary Figure S3**

**
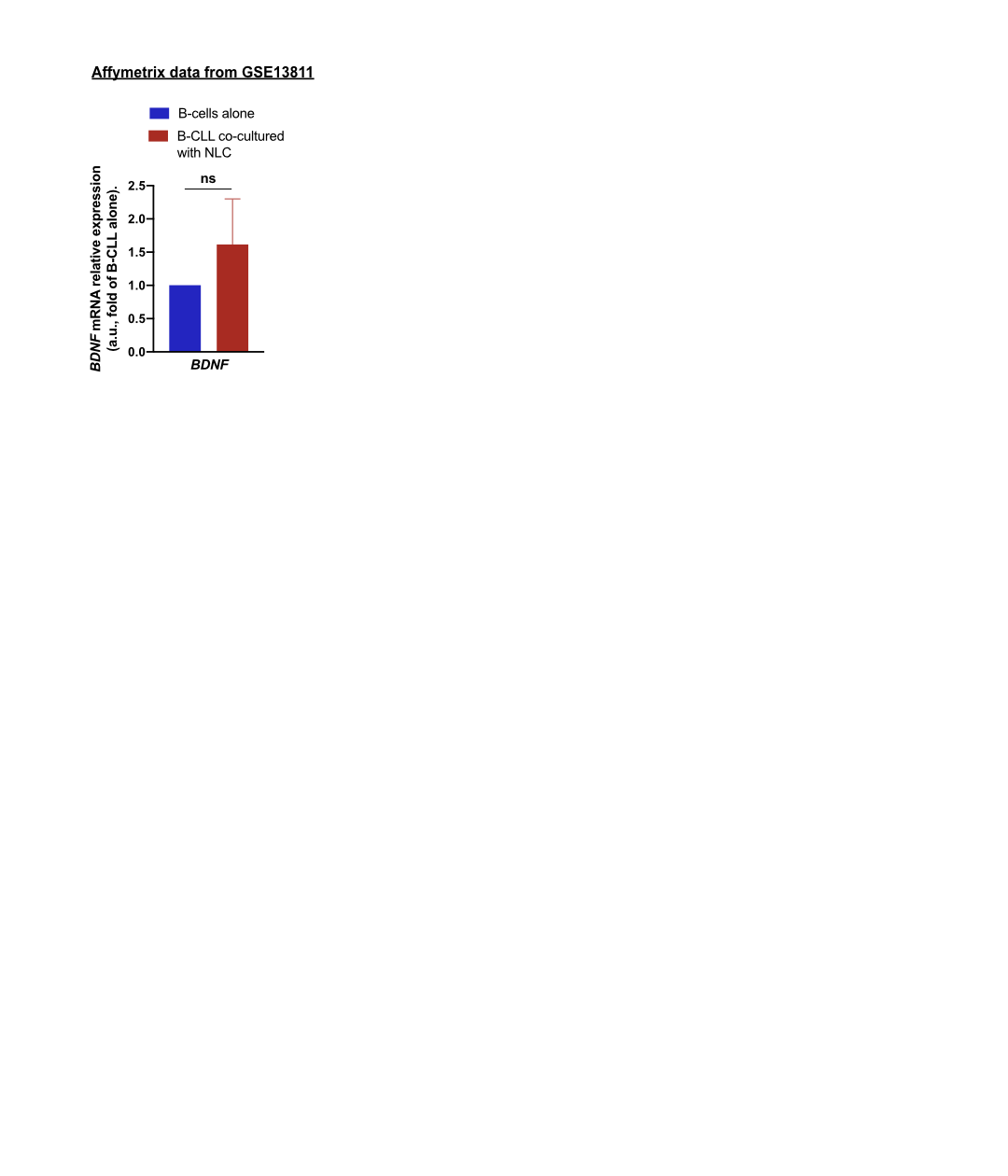
**

**Affymetrix analysis of *BDNF* mRNA from GSE13811**

Relative expression of *BDNF* mRNA in B-CLL cells co-cultured for 14 days with autologous NLC (n = 9), compared with B-CLL cells alone (n = 9) purified from the same patient’s blood. Analyzed using the Affymetrix Human Genome U133 Plus 2.0 Array (dataset GSE13811). Data are presented as mean ± SEM from at least three independent experiments with ns=not significant.

**Supplementary Figure S4**


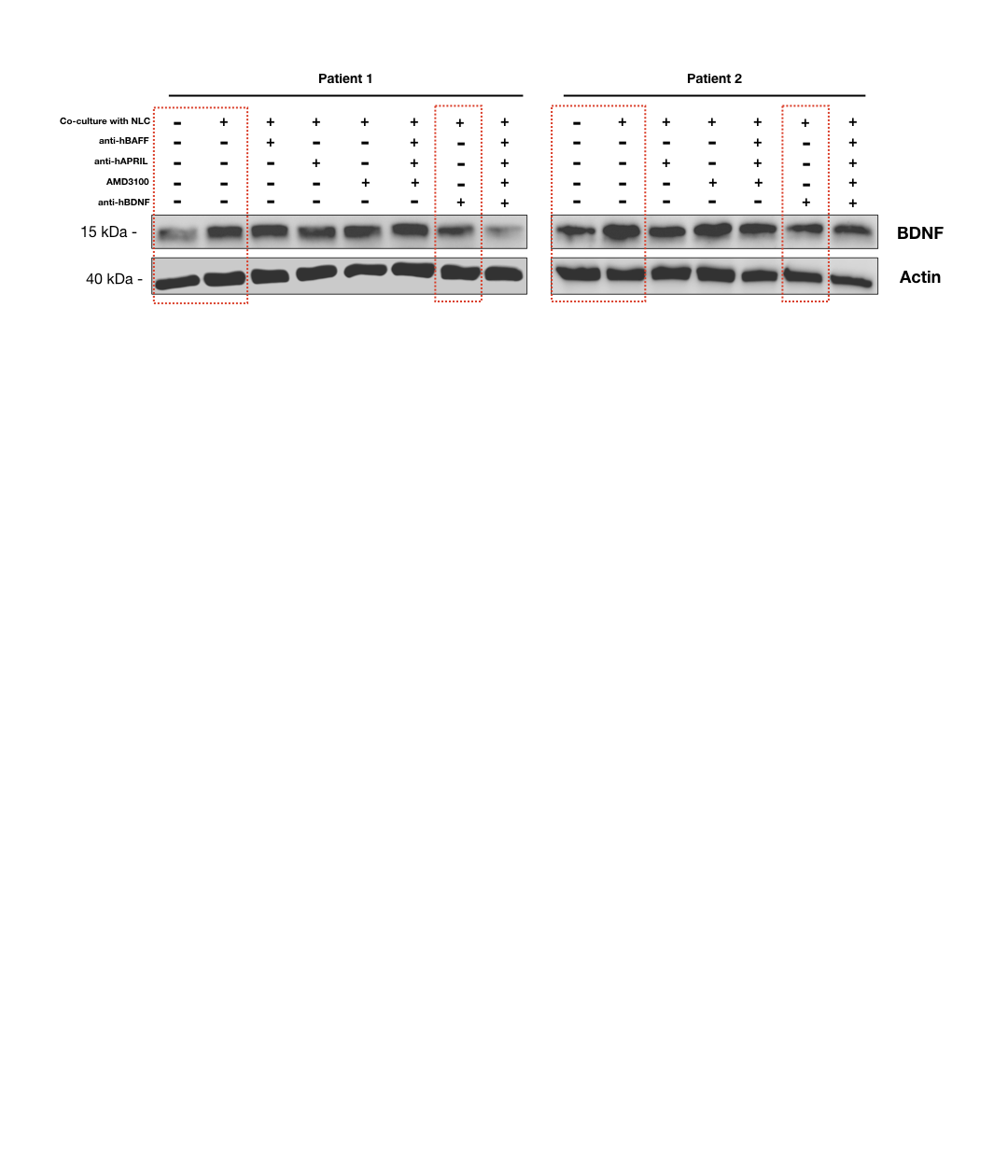
**Whole western blot membranes corresponding to Figure 2 e.**

Representative western blot analysis (whole membranes) of p-Src and Bcl-2 in B-CLL cells isolated from two independent patients. Cells were cultured for 72h alone, with autologous NLC, or with autologous NLC and single or combined inhibition of BAFF (anti-hBAFF, 100 ng/mL), APRIL (anti-hAPRIL, 500 ng/mL), CXCR4 (AMD3100, 0.5 µg/mL), and BDNF (anti-hBDNF, 200 ng/mL). Red rectangles highlight the bands presented and analyzed in Figure 2e.

**Supplementary Figure S5**

**
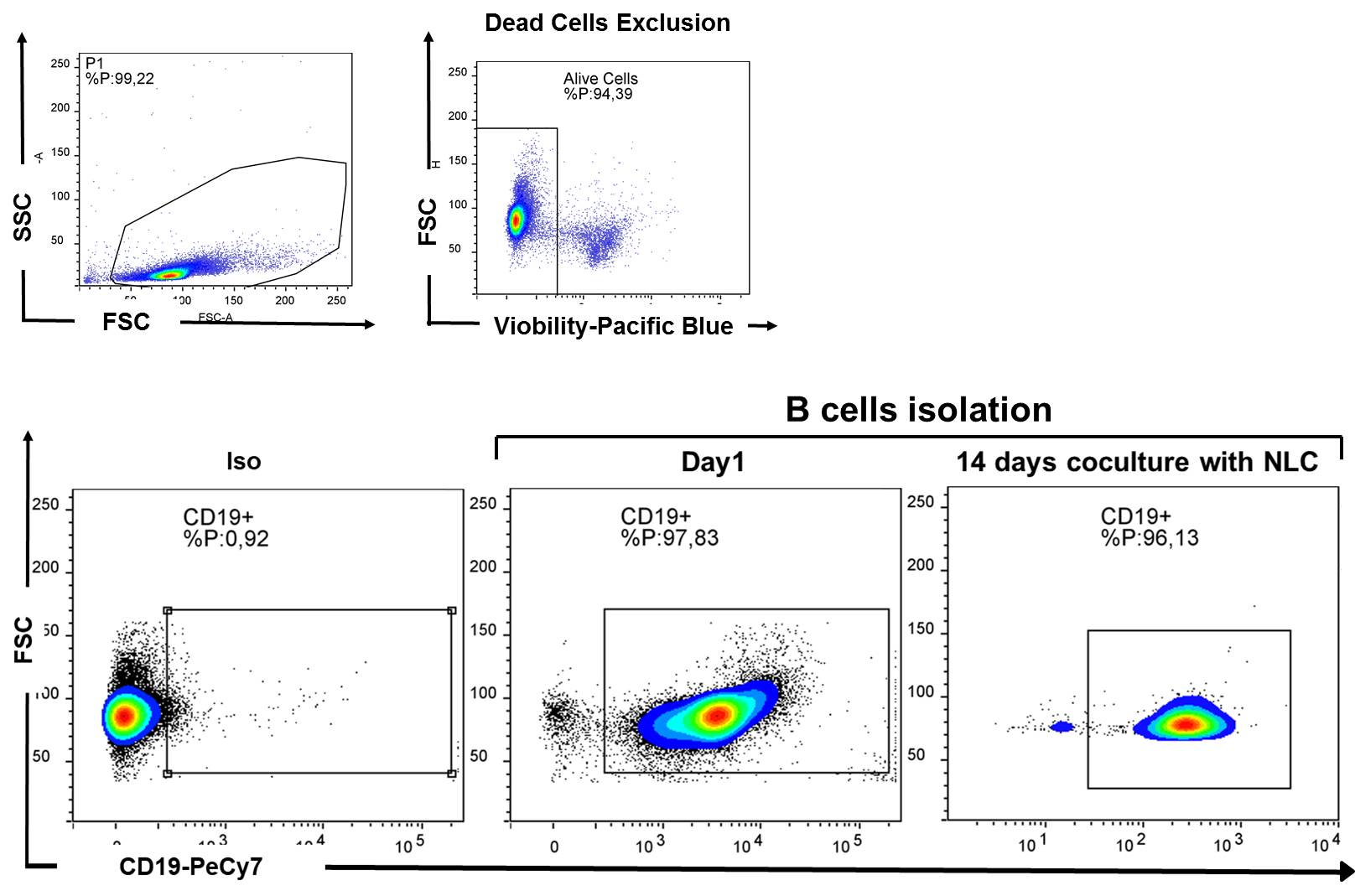
**

**B cell purity**

Flow cytometry analysis of CD19 expression gated on alive cells (top panel), after B cell isolation using negative selection kit (B-CLL Cell Isolation Kit, human, Miltenyi Biotec). B cell purity was assessed by CD19 percentages after isolation from PBMC of CLL patients, forthwith blood collection (Day1), and on floating cells following 14 days co-cultured with NLC (bottom panel).

**Supplementary Figure S6**

**
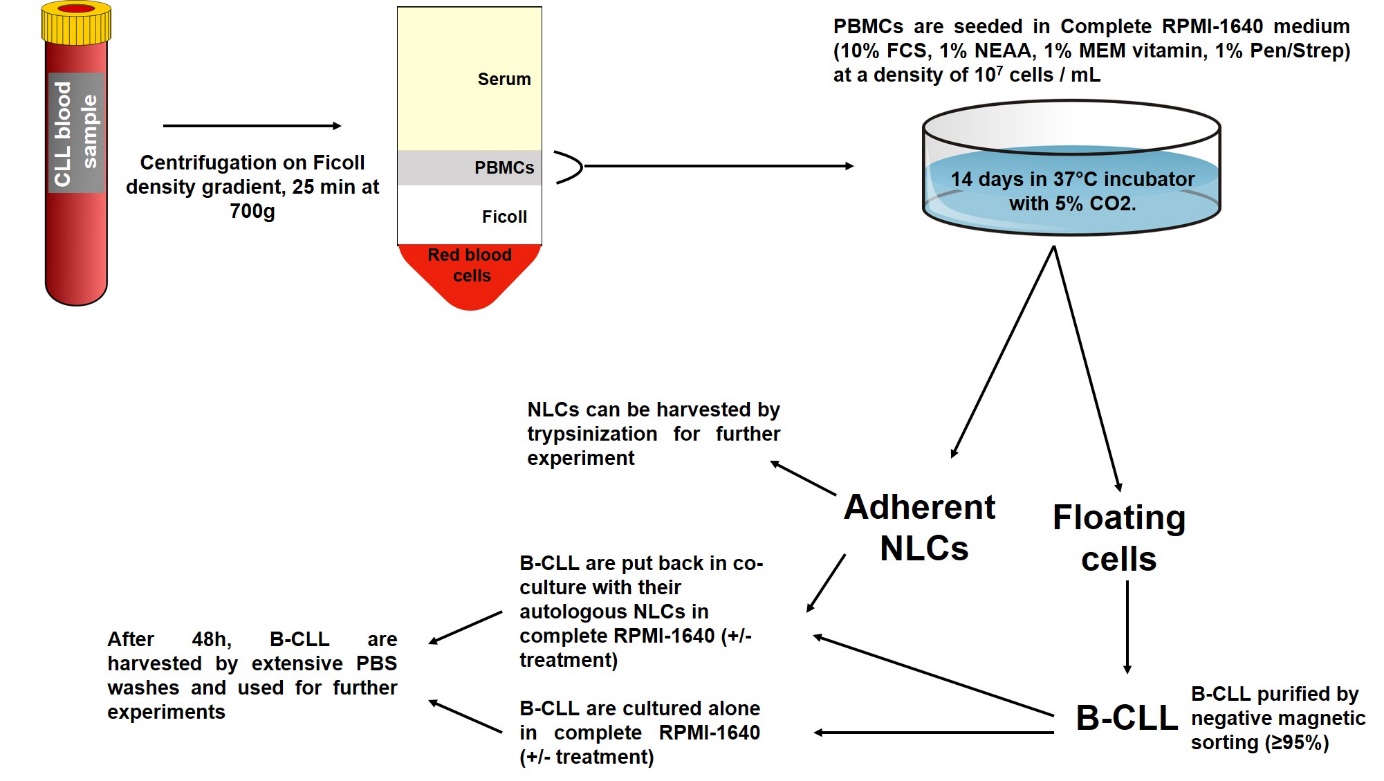
**

***Experimental procedure for B-CLL and NLC purification and co-cultures***

**Supplementary Figure S7**

**Full-length western blot membranes**

**
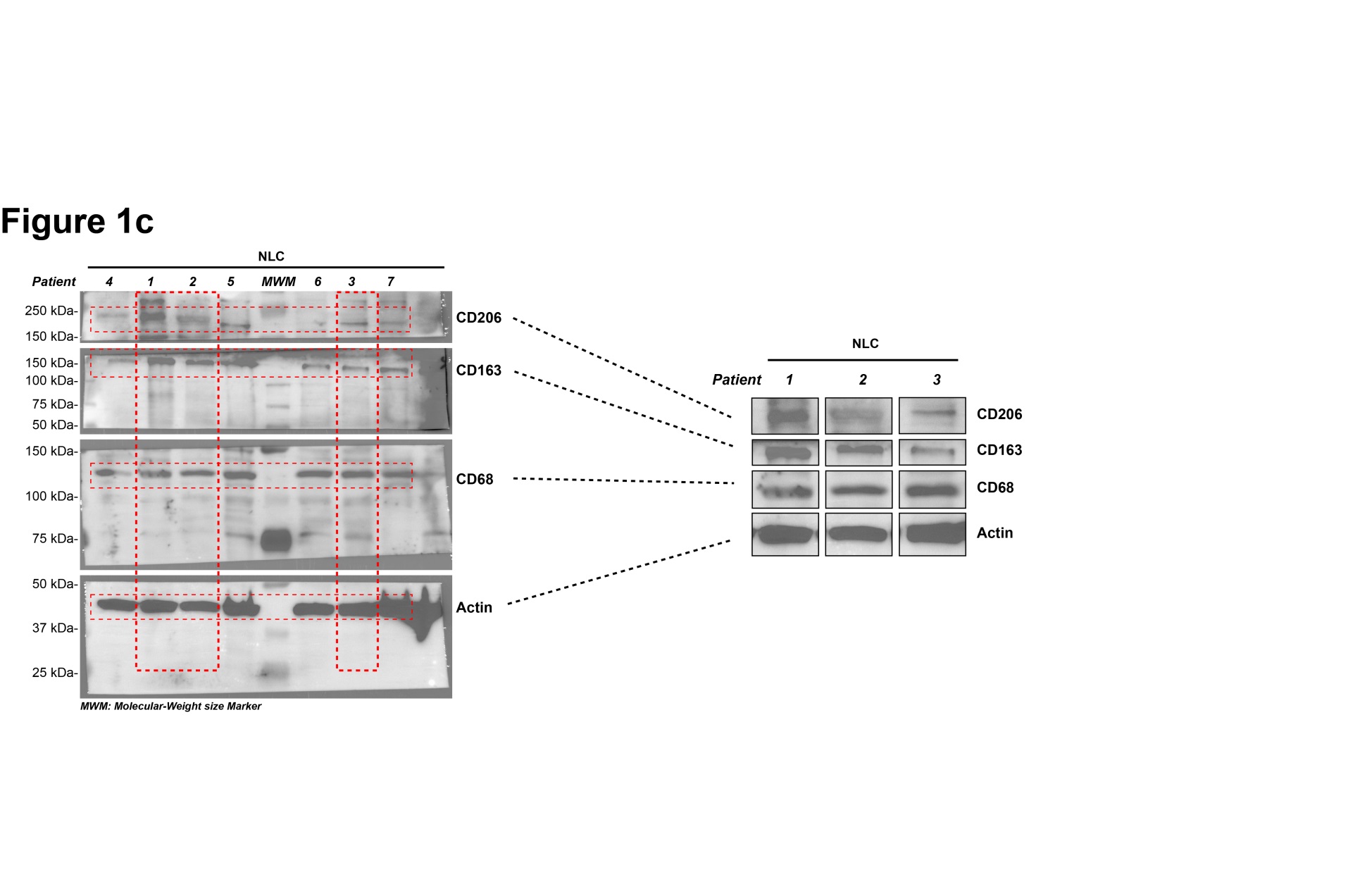
**

**
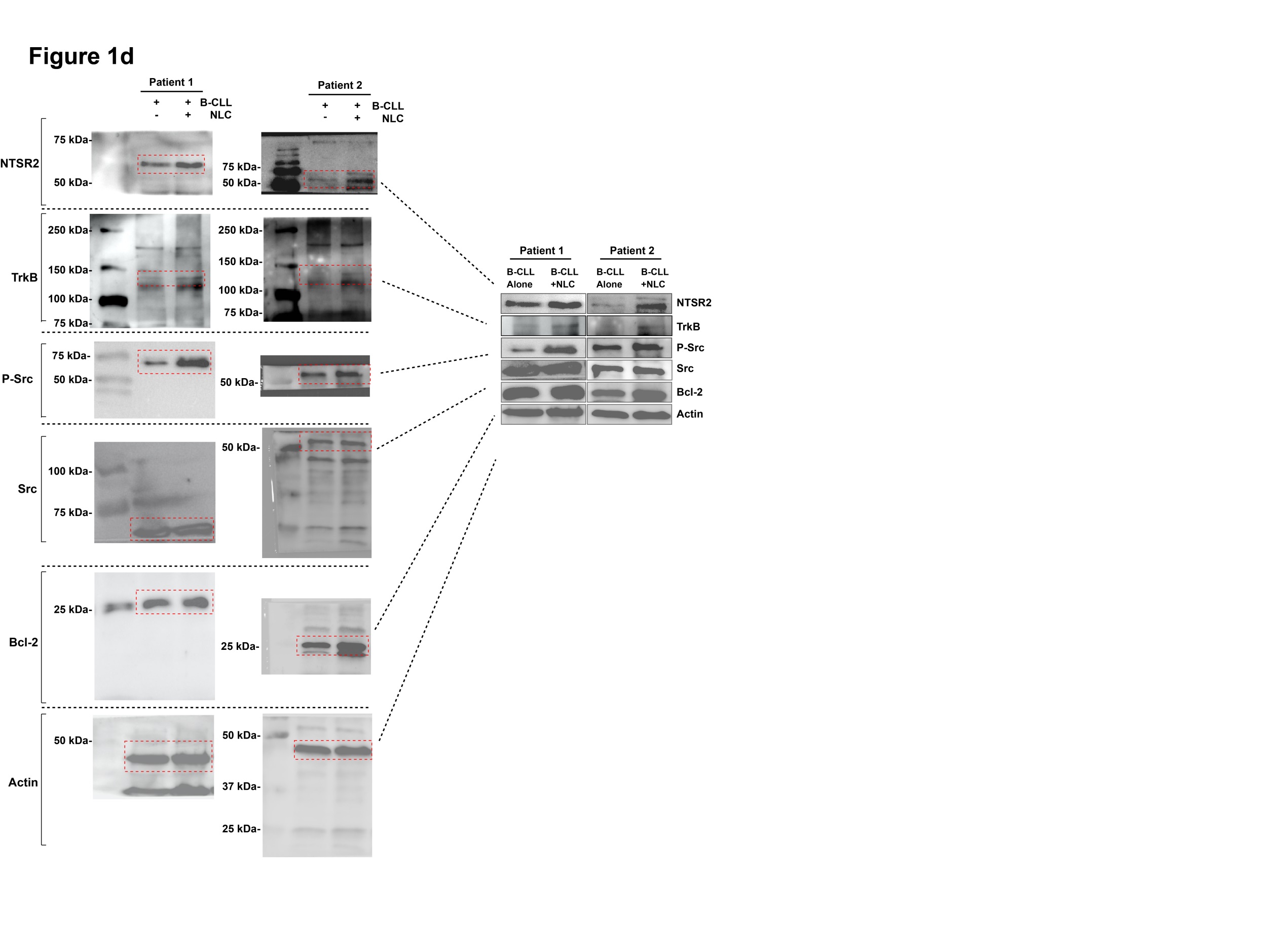
**

**
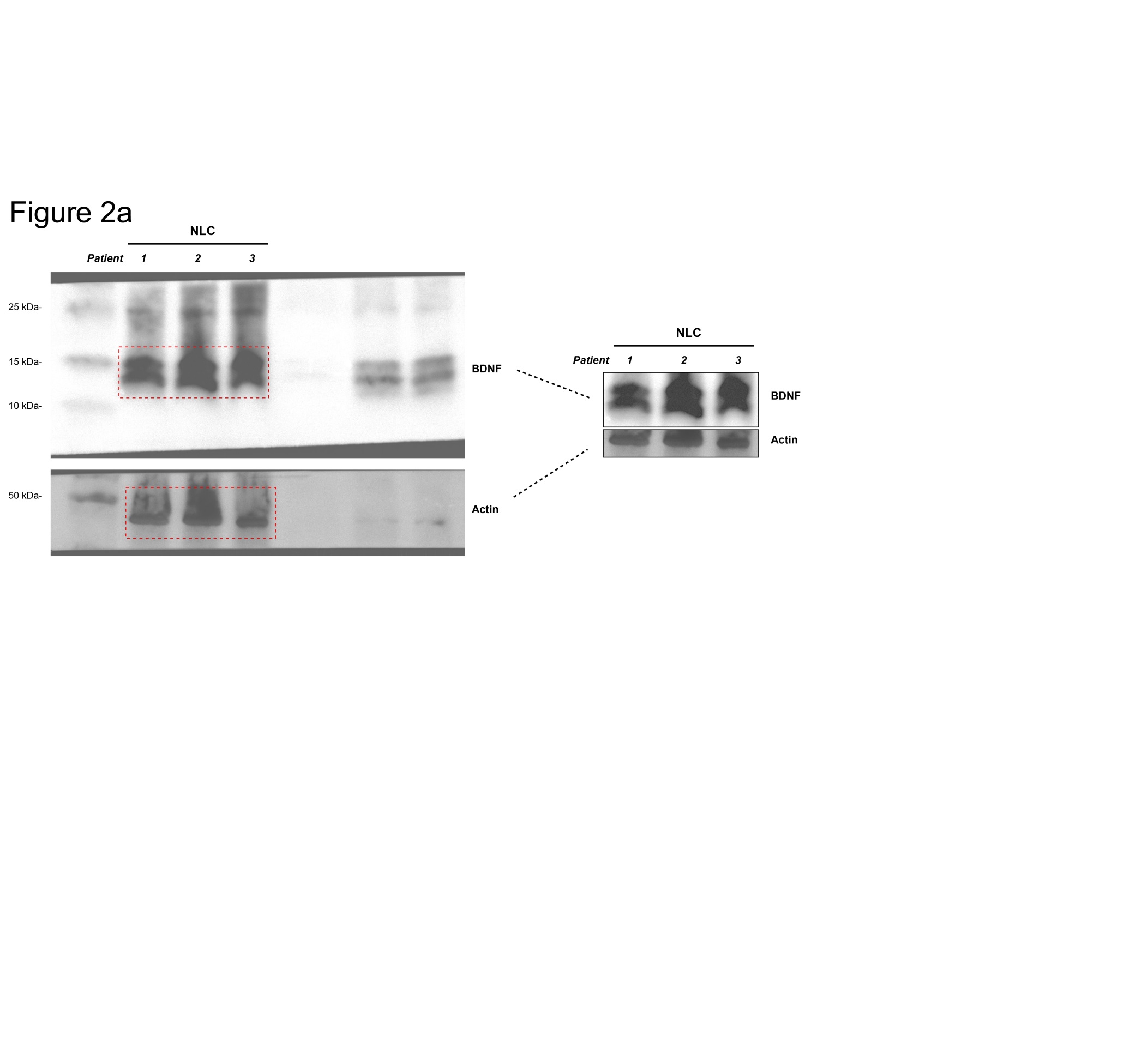
**

**
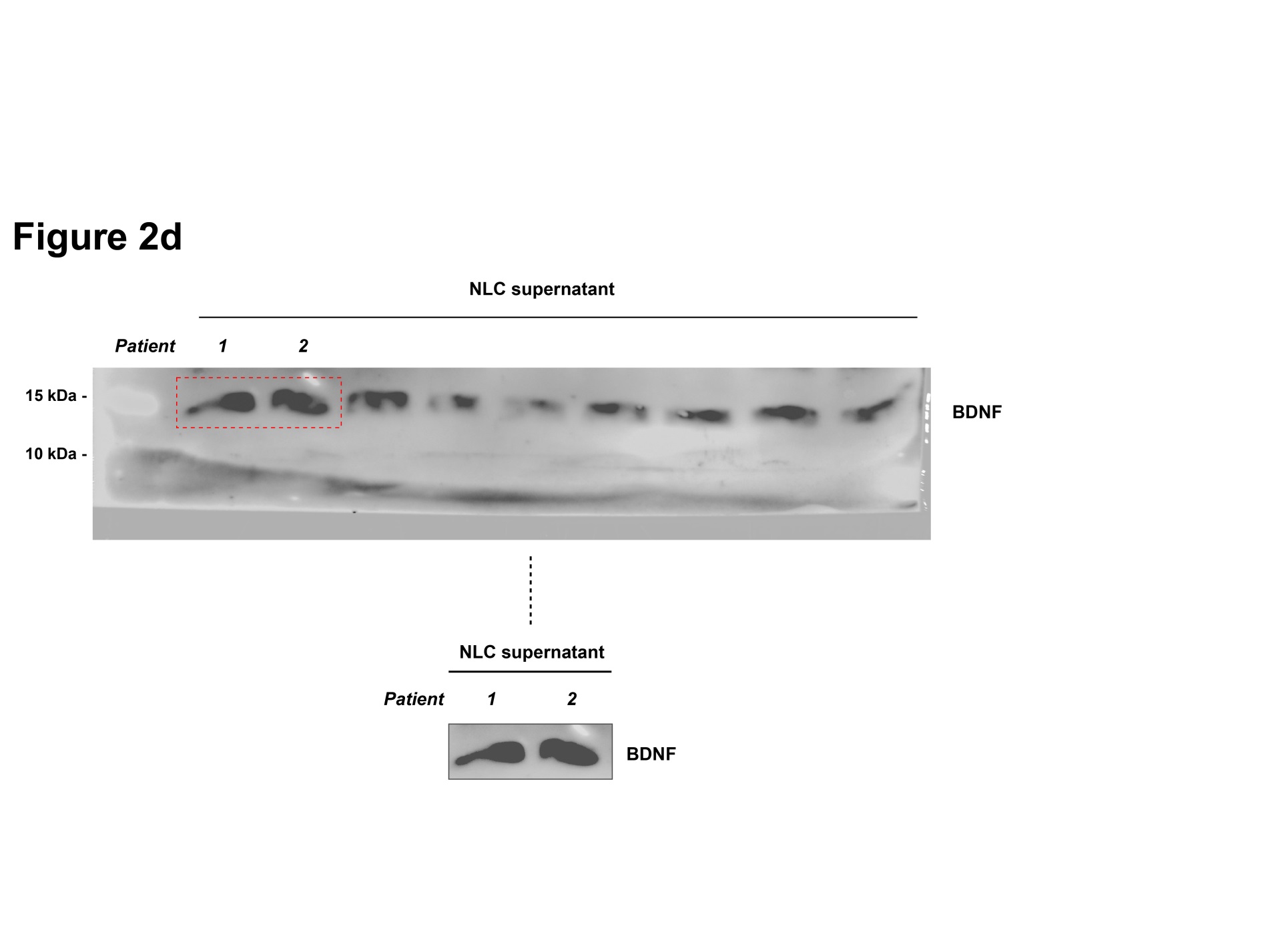
**

**
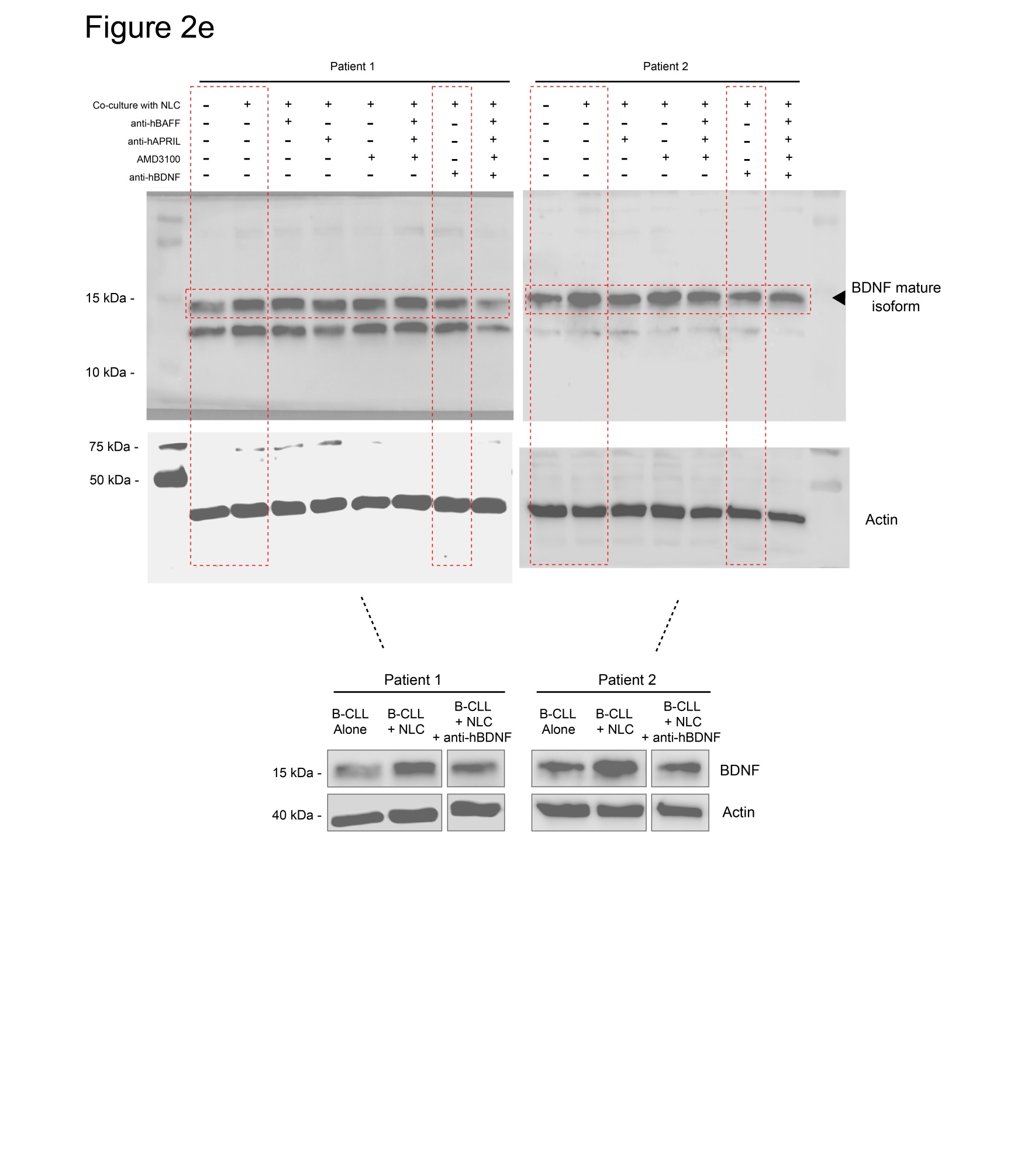
**

**
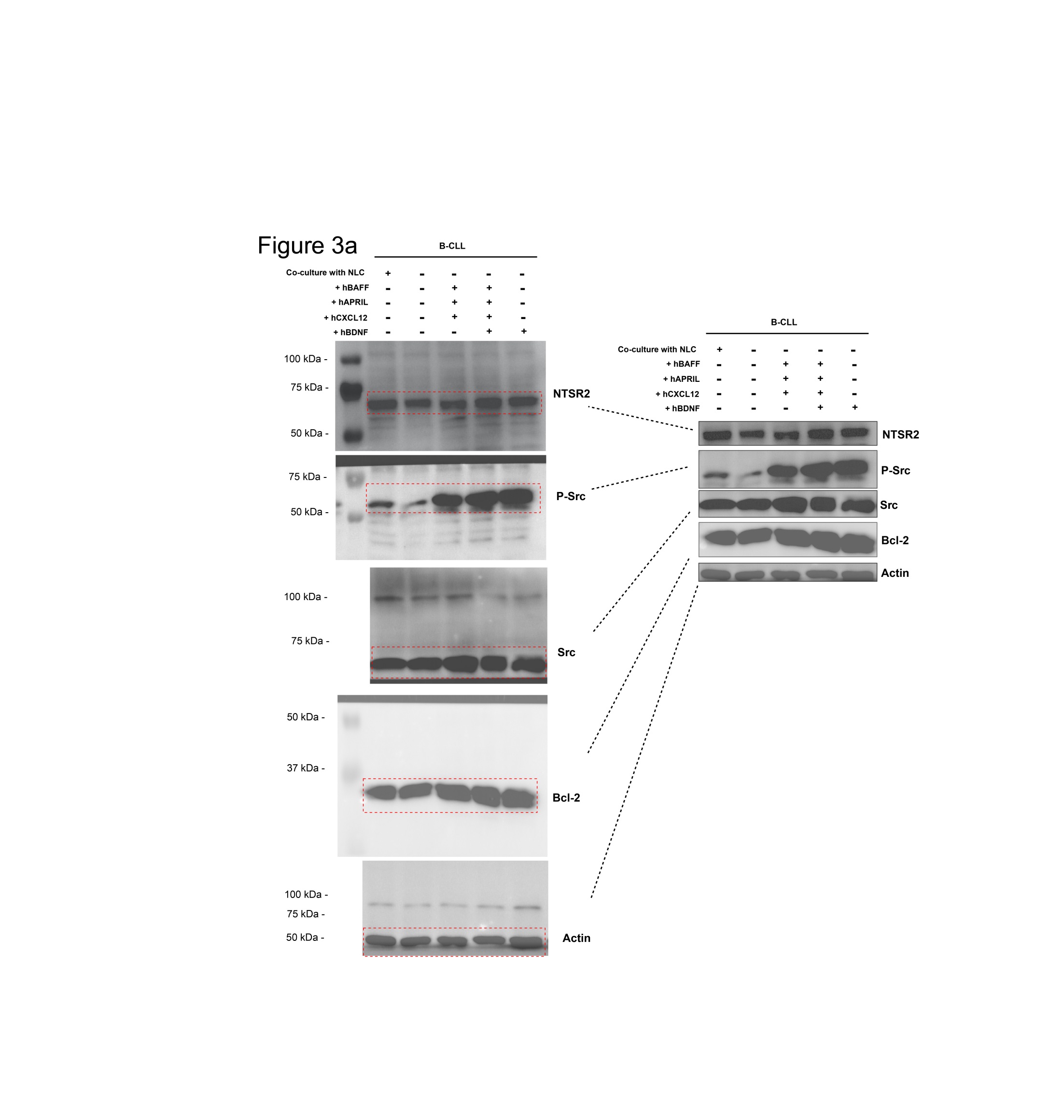
**

**
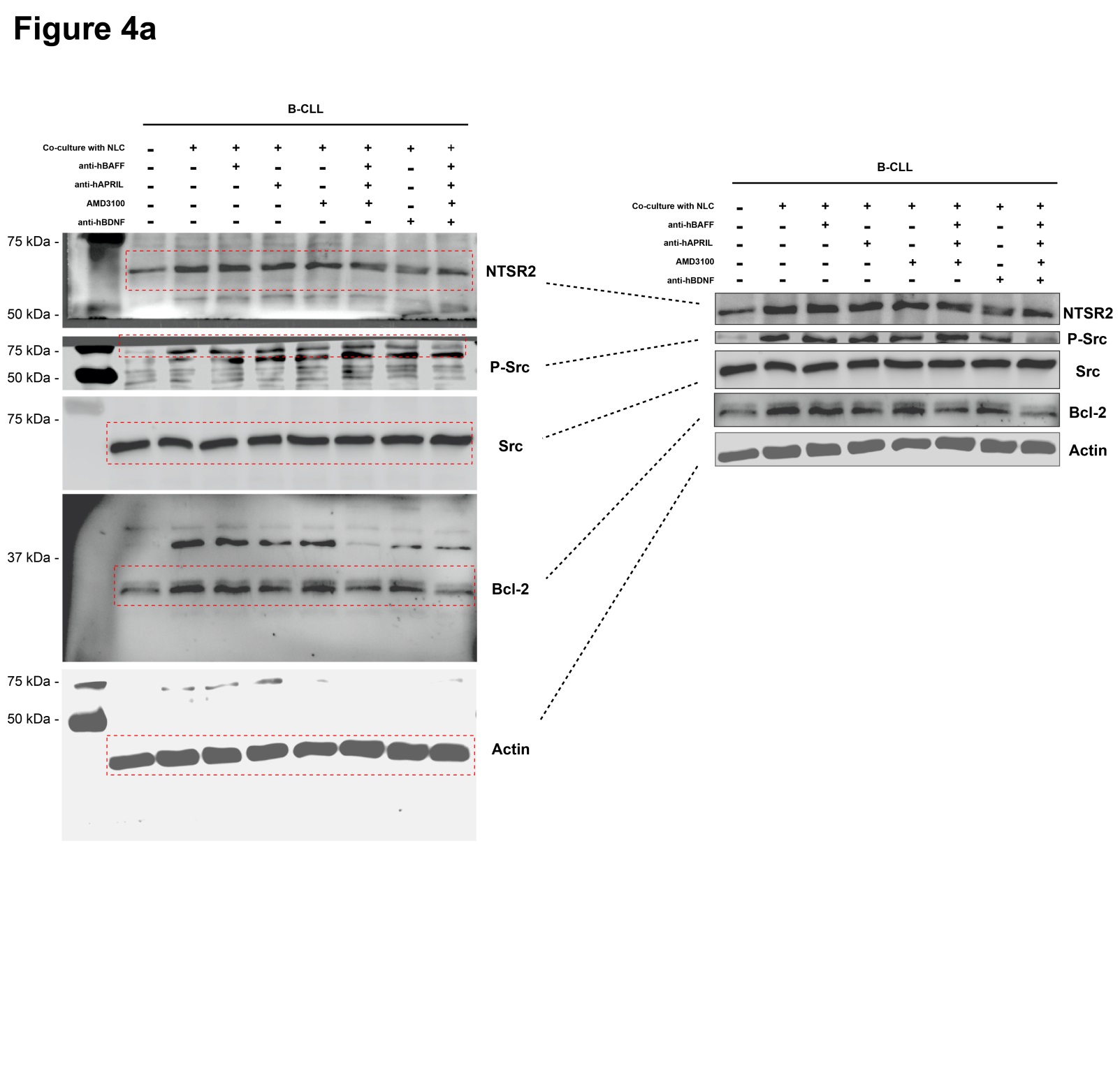
**


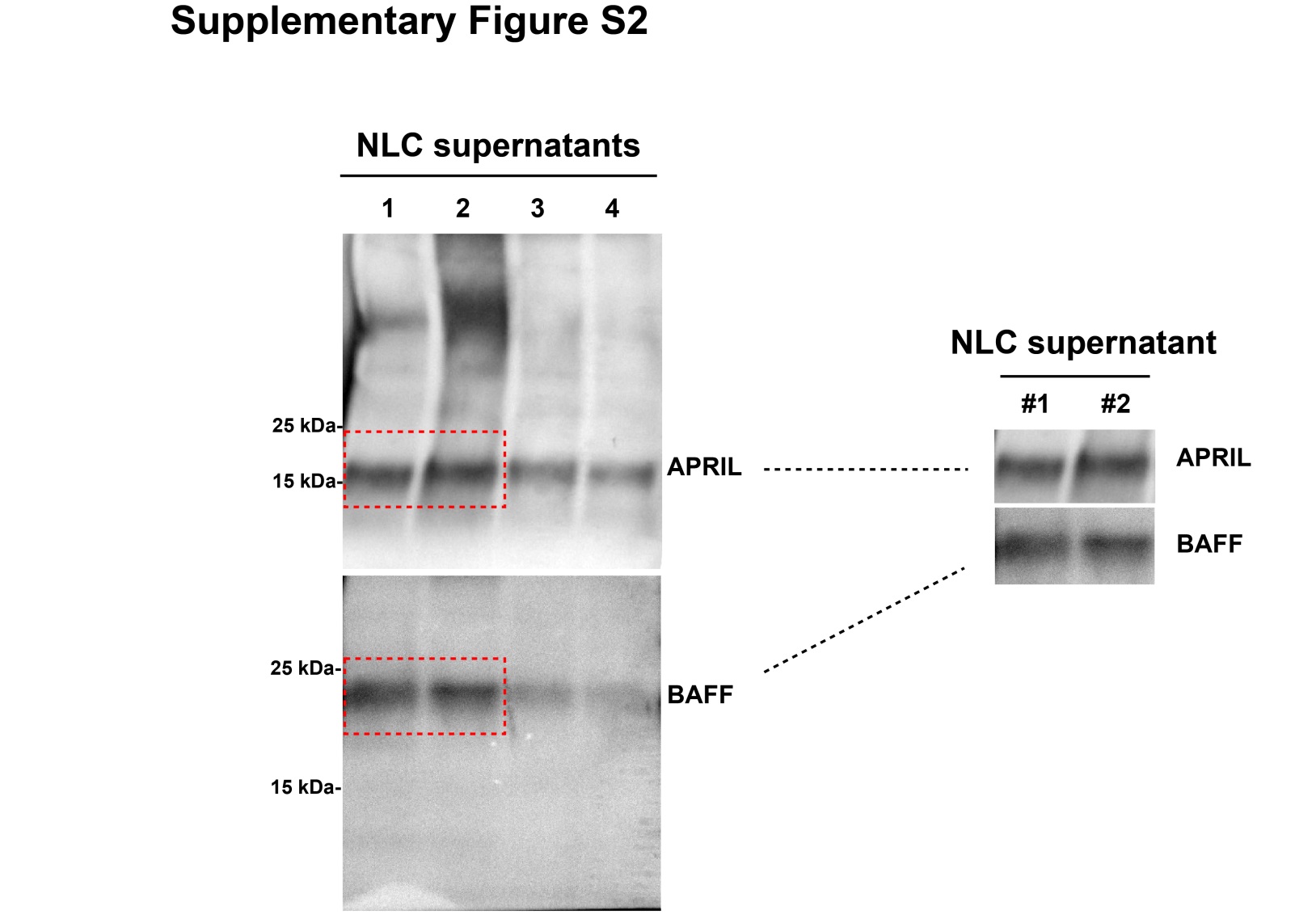


1-4: APRIL and BAFF detections in NLC supernatants from 4 CLL patients.
